# Loss of ARID1A Expression as a Favorable Prognostic Factor in Early-Stage Grade 3 Endometrioid Endometrial Carcinoma Patients

**DOI:** 10.3389/pore.2021.598550

**Published:** 2021-03-25

**Authors:** Mayumi Kobayashi Kato, Hiroshi Yoshida, Yasuhito Tanase, Masaya Uno, Mitsuya Ishikawa, Tomoyasu Kato

**Affiliations:** ^1^ Department of Gynecology, National Cancer Center Hospital, Tokyo, Japan; ^2^ Department of Diagnostic Pathology, National Cancer Center Hospital, Tokyo, Japan

**Keywords:** endometrioid endometrial carcinoma, grade 3, prognosis, ARID1A, p53, mismatch repair

## Abstract

**Introduction:** High-risk patients with grade 3 endometrioid endometrial carcinoma (G3EEC) who require adjuvant therapy have not been clearly identified. Therefore, the current study aimed to investigate the prognostic impact of ARID1A, p53, and mismatch repair (MMR) protein expressions, previously reported as prognosticators in some gynecological cancers, in patients with early-stage G3EEC.

**Methods:** A total of 67 patients with pathologically confirmed early-stage G3EEC diagnosed between 1997 and 2020 were identified; none received adjuvant chemotherapy. The recurrence-free survival (RFS) and overall survival (OS) were estimated using the Kaplan-Meier method and compared with a log-rank test. The protein expressions of ARID1A, p53, and MMR were examined via immunohistochemistry, and the associations between these biomarkers and clinical outcomes were evaluated.

**Results:** Recurrence was observed in 9 (13%) of the 67 patients with early stage G3EEC. The respective 5-years RFS and OS rates were 87.7% and 93.7%, and 68.6% and 85.7%, respectively for stages I and II. Multivariate analysis showed significantly longer RFS among patients with ARID1A loss (hazard ratio = 8.7; 95% CI, 1.09–69.6, *p* = 0.04). No significant differences were observed in RFS and OS of patients according to p53 and MMR expression status.

**Conclusion:** ARID1A expression status was a prognosticator for patients with early stage G3EEC without adjuvant therapy, whereas p53 and MMR expression status showed no impact on survival outcomes. ARID1A may become a useful biomarker for stratification of adjuvant treatment for early stage G3EEC patients.

## Introduction

The prevalence of endometrial cancer has been increasing worldwide, with over 380,000 new cases diagnosed in 2018 [1]. In Japan, endometrial cancer is the most commonly observed gynecologic malignancy [2]. Endometrial cancers have been classified as type 1 or type 2 on the basis of the associated risk factors and prognosis [2]. In contrast to type 1, type 2 endometrial cancer is typically not estrogen-driven, and clinically behaves more aggressively.

Grade 3 endometrioid endometrial carcinoma (G3EEC) is conventionally categorized as a type 2 endometrial cancer along with uterine serous carcinoma (USC) and clear cell carcinoma (CCC). Some case series reported similar survival outcomes between G3EEC, USC, and CCC [3–8]. However, in other studies, particularly a retrospective review that used data obtained from the Surveillance, Epidemiology, and End Results Program, patients with G3EEC had a significantly better overall survival (OS) rate than that of patients with USC or CCC [9–11]. Owing to these conflicting results, adjuvant therapy implementation for early-stage G3EEC is controversial; therefore, it is important to clarify the prognostic factors of early stage G3EEC.

Negative prognostic factors of endometrial carcinoma include non-estrogen dependent tumors (type 2 endometrial cancer), elderly age, and advanced International Federation of Gynecology and Obstetrics (FIGO) stage [12]. Additionally, the expression status of ARID1A, p53, and mismatch repair (MMR) proteins can be independent prognosticators [13, 14].

Significant differences were observed between FIGO stages I-IV clear cell and endometrioid subtypes of ovarian and endometrial cancer according to the protein expression status of ARID1A. Several studies reported that ARID1A mutations correlated with a favorable survival outcome [15, 16]. In addition, aberrant p53 expression was significantly associated with a higher tumor grade and shorter OS [13]. Moreover, MMR deficiency (dMMR) was associated with elderly age, higher tumor grade (G3), and advanced stage (II-IV), whereas MLH1 promoter hypermethylation predicted a shorter disease-specific survival [14].

However, these studies included all histologic types and stages of endometrial cancer. Only a few studies evaluated the association between prognosis and expression of ARID1A, p53, and MMR, specifically for early-stage G3EEC patients.

In the current study, we retrospectively reviewed patients with early-stage G3EEC and performed immunohistochemistry (IHC) for ARID1A, p53, and MMR proteins. Notably, we could evaluate the baseline recurrence risk in our patients because none of them received adjuvant treatment during the study period due to the lack of compelling evidence in its favor. The study aimed to clarify the prognostic impact of the expression status of ARID1A, p53, and MMR proteins on the outcomes of patients with early-stage G3EEC.

## Methods

### Patient Demographic Characteristics

The study was approved by the Institutional Review Board of the National Cancer Center in Japan (#2016-260) and conducted in accordance with the Declaration of Helsinki. The requirement for informed consent was waived due to the retrospective nature.

Patients with pathologically confirmed stage I and stage II (per the 2008 FIGO classification) G3EEC diagnosed between 1997 and 2020 were identified from the tumor registry database of our institution. All tumors were surgically resected by gynecologic oncologists. All surgical staging procedures, including total hysterectomy, bilateral salpingo-oophorectomy, and pelvic lymph node sampling, were performed via laparotomy. At least two gynecologic pathologists confirmed the histopathological diagnosis of G3EEC and absence of lymph node metastases. Although the Japanese treatment guidelines for endometrial cancer recommend adjuvant chemotherapy for stage IA and IB/II G3EEC, there is no strong evidence supporting this treatment strategy [17]. Thus, the patients included in the present study did not receive adjuvant treatment. The clinicopathological data were collected via a retrospective review of medical charts.

The follow-up for most patients comprised vaginal inspections, Pap smear cytology, and radiological examination including chest radiography every 3-6 months, and computed tomography (CT) of the chest, abdomen, and pelvis every 6 months for the first 2 years. During the third and fourth years of follow-up, patients underwent vaginal examinations, two Pap smears, and annual radiological examinations. After five years of follow-up, patients underwent vaginal inspections and Pap smears annually, and those with suspected G3EEC recurrence underwent CT of the chest to pelvis, magnetic resonance imaging, and histological examination.

### Immunohistochemistry Analysis and Interpretation

All the surgically resected specimens were fixed in 10% neutral-buffered formalin for 24–72 h and embedded in paraffin. One representative whole 4 μm-thick section was analyzed using IHC. The following antibodies were used for IHC on the representative slides for each case: anti-p53 (DO7, pre-diluted; Dako, Glostrup, Denmark); anti-ARID1A (polyclonal, 1:2,000 dilution; Sigma, St. Louis, MO, United States); anti-hMSH6 (SP93, 1:200 dilution; Spring Bioscience, CA, United States); and anti-hPMS2 (A16-4, 1:200 dilution; Biocare Medical, CA, United States) antibodies. We performed all the IHC tests using a Dako autostainer (Dako, CA, United States) according to the manufacturer’s recommendations. After deparaffinization, tissue sections were stained using the antibodies described above, and then counterstained with hematoxylin.

Aberrant p53 staining pattern was defined as a strong and diffuse nuclear staining pattern (>70% of carcinoma cells) or completely negative (“null pattern”) staining of carcinoma cells with appropriate staining of surrounding non-tumor cells as an internal positive control. A weak and heterogeneous staining pattern of tumor cells was classified as the wild-type pattern.

We assessed ARID1A expression by immunohistochemistry and categorized the results into 3 classes, namely, retained expression (positivity in almost all tumor cells), homogenous loss of expression (negativity in almost all or >90% of tumor cells), and heterogeneous loss of expression (regional negativity with 10–90% of ARID1A-negative tumor cells). Both, homogenous and heterogenous loss of ARID1A expression were regarded as ARID1A loss.

**FIGURE 1:**
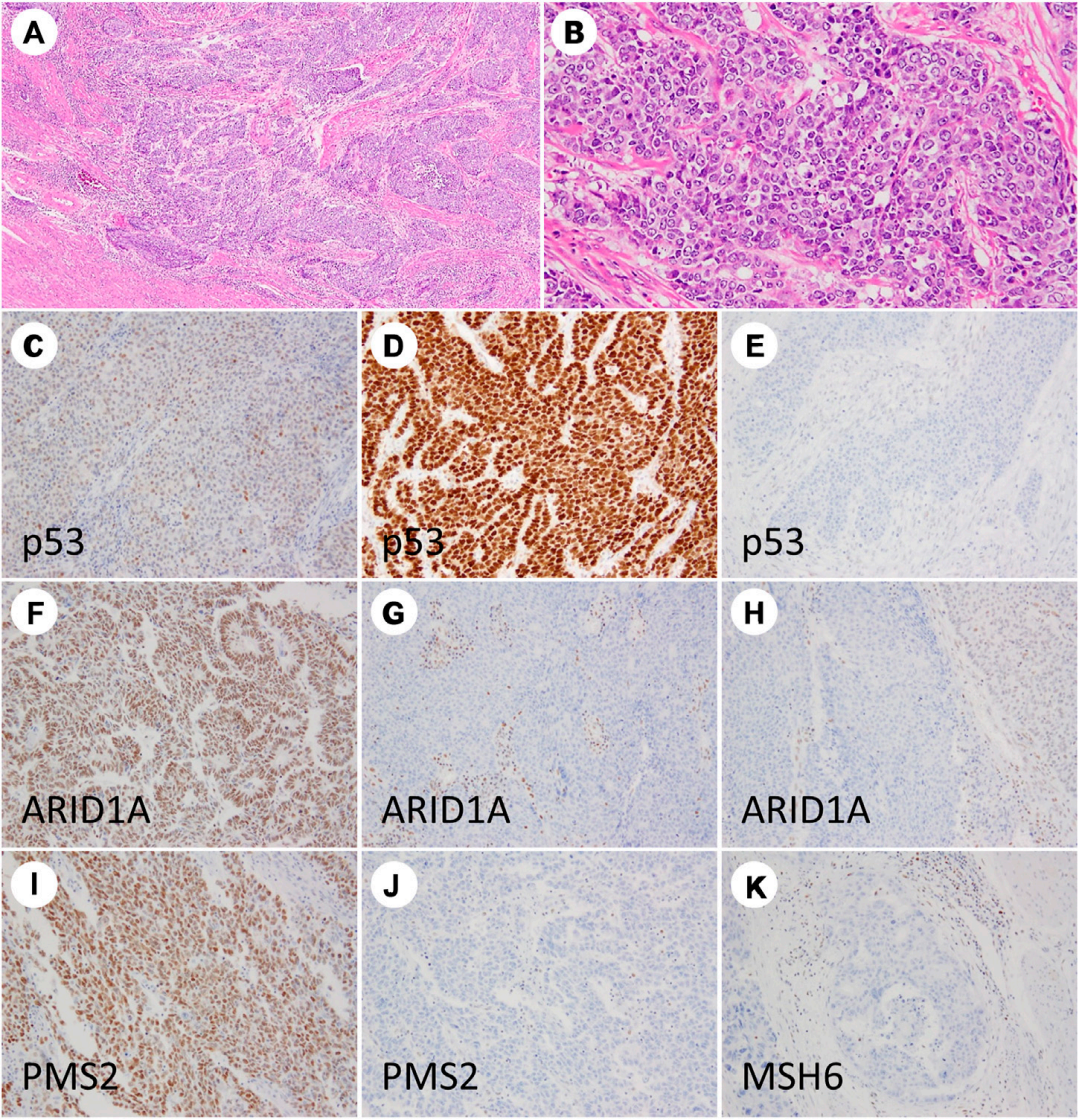
Immunohistochemistry staining results of grade 3 endometrioid endometrial carcinoma. The tumor is dominantly composed of solid nests **(A,B)**. TP53 showing wild-type **(C)**, diffuse strong **(D)**, and null patterns **(E)**. ARID1A showing intact staining **(F)**, complete loss **(G)**, and partial loss **(H)**. PMS2 nuclear staining is retained **(I)** and lost **(J)**. MSH6 staining is lost **(K)**.

IHC for PMS2 and MSH6 alone can reportedly replace a four antibody panel (comprising MLH1, MSH2, MSH6, and PMS2) for dMMR screening [18]; therefore, in the present study, MMR-deficient status was defined as the complete loss of nuclear staining for PMS2 and/or MSH6 proteins. Adjacent normal mucosa, stromal cells, and inflammatory cells with intact nuclear staining served as internal positive controls. Representative images of IHC for p53, ARID1A, and MMR proteins are depicted in Figure 1.

### Statistical Analysis

The follow-up time and time to events were measured from the date of surgery to the last known visit, confirmation of recurrence, or death. Recurrence-free survival (RFS) was defined as the time from the date of surgery to the date of the first recurrence. OS was defined as the time from the date of surgery to the date of death due to any cause. Survival values were estimated using the Kaplan-Meier method and compared with log-rank tests. Independent prognostic factors for RFS were determined using Cox proportional hazards regression models. All analyses were performed using the JMP14 software program (SAS Institute Japan Ltd., Tokyo, Japan).

## Results

### Patient Demographic Characteristics

Between 1997 and 2020, a total of 113 patients were diagnosed with G3EEC. All surgical specimens were histologically reviewed, and immunostaining was performed for 110 patients with available specimens.

Among the 113 patients with G3EEC, 67 were identified with FIGO stage I and II disease. None of the patients with early-stage disease received adjuvant treatment; the median age was 58 years (range, 43–80 years). The clinicopathological features of the patients with stage I–II disease are summarized in Table 1.

**TABLE 1:** Characteristics of patients with early stage grade 3 endometrioid endometrial carcinoma.

Characteristic		No (%) (n = 67)
Stage (FIGO 2008)	IA	35 (52)
	IB	25 (37)
	II	7 (10)
Age, years	<65	47 (70)
	≥65	20 (30)
BMI	<30	62 (93)
	≥30	5 (7)
Depth of invasion	Endometrium	5 (7)
	Myometrium <50%	31 (46)
	Myometrium ≥50%	31 (46)
Cervical stromal involvement	Present	7 (10)
	Absent	60 (90)
LVSI	Present	42 (63)
	Absent	25 (37)
Pelvic cytology	Positive	8 (12)
	Negative	59 (88)

BMI, body mass index; FIGO, international federation of gynecology and obstetrics; LVSI, lymphovascular space invasion

### Treatment and Prognosis of Patients With Early-Stage G3EEC

All patients with early-stage G3EEC underwent total hysterectomy and bilateral salpingo-oophorectomy. Pelvic lymph node sampling was performed in 60 patients (89.6%). Seven patients did not undergo lymph node sampling due to their advanced age and complications. The median number of removed lymph nodes was 29 (range, 1–58). The median follow-up time for patients with early-stage G3EEC was 65 months (range, 3–182 months). Recurrence was observed in nine patients (13.4%), and the median time to recurrence was 22 months (range, 9–45 months). Recurrences in the vagina, lymph nodes, and other distant sites were observed in one, three, and five patients, respectively.

The clinicopathological features, treatment, and prognosis of the patients with recurrence are summarized in Table 2. The five-year RFS rates for patients with stage I and II disease were 87.7 and 68.6%, respectively. The five-year OS rates for patients with stage I and II disease were 93.7 and 85.7%, respectively. The median RFS and OS were 63.2 months (range, 3–220 months) and 65.6 months (range, 3–227 months), respectively.

**TABLE 2:** Clinicopathological characteristics, treatment, and outcomes of patients with recurrence of early-stage grade 3 endometrioid endometrial carcinoma.

Case	Age	Stage	Tumor size (mm)	Depth of invasion	LVSI	p53 IHC	ARID1A IHC	MMR status	Site of recurrence	Treatment for recurrence	DFS (mo)	Status at last follow-up
1	63	IA	33	<1/2	Present	Wild type	Lost (homo)	deficient (PMS2)	Bone	RT + Chemotherapy	12	LOF, 17 months
2	57	II	35	≥1/2	Absent	Wild type	Retained	Proficient	lung (44mo), abdominal mass (96mo)	Surgery + Chemotherapy	44	NED, 182 months
3	65	IB	120	≥1/2	Present	Wild type	Retained	Proficient	PAN	Surgery + Chemotherapy	13	NED, 69 months
4	70	II	66	≥1/2	Present	Diffuse	Retained	Proficient	PLN	MPA	17	DOD, 32 months
5	67	IB	40	≥1/2	Present	Wild type	Retained	Proficient	peritoneum + pleura	Chemotherapy	21	DOD, 40 months
6	69	IB	15	≥1/2	Present	Wild type	Retained	Proficient	Lung	Chemotherapy	27	DOD, 47 months
7	54	IB	70	≥1/2	Present	Wild type	Retained	Deficient (PMS2)	Vagina	RT	6	NED, 45 months
8	54	IB	70	≥1/2	Present	Diffuse	Retained	Proficient	Liver, PAN	Chemotherapy	10	NED, 23 months
9	71	IB	40	≥1/2	Present	Diffuse	Retained	Proficient	Lung	Chemotherapy	9	AWD, 9 months

LVSI, lymphovascular space invasion; ARID1A, AT-rich interaction domain 1A; MMR, mismatch repair; DFS, disease-free survival; LOF, lost to follow-up; NED, no evidence of disease; PAN, para-aortic lymph node; PLN, pelvic lymph node; MPA, medroxyprogesterone acetate; DOD, died of disease

### Immunohistochemistry for ARID1A, p53, and MMR Proteins

The results of IHC staining of early stage G3EEC are shown in Table 3. An aberrant expression pattern of p53 (overexpression or complete absence) was observed in 21 of 67 cases (31.3%), loss of MMR protein expression was observed in 40.3% of cases, isolated PMS2 loss was observed in 22 of 27 dMMR cases (81.5%), and loss of ARID1A expression was observed in approximately half of the patients (50.7%). There were no significant correlations between ARID1A, p53, and MMR expression status. Moreover, the expression status of these biomarkers was not correlated with clinicopathological features such as tumor size (greatest dimension), depth of myometrial invasion, cervical stromal invasion, lymphovascular invasion, and intraoperative peritoneal cytology.

**TABLE 3:** Number of patients showing immunohistochemical staining of early-stage grade 3 endometrioid endometrial carcinoma.

Marker	Staining pattern	No (%) (n = 67)
p53	Aberrant pattern	21 (31)
	Diffuse pattern	20 (29)
	Null pattern	1 (1)
	Wild-type pattern	46 (69)
ARID1A	Retained	33 (49)
	Lost	34 (51)
	Homogenous pattern	22
	Heterogenous pattern	12
MMR	Proficient	40 (60)
	Deficient	27 (40)
	PMS2	22
	MSH6	5

ARID1A: AT-rich interaction domain 1A, MMR: mismatch repair.

### Association Between Prognosis and The ARID1A, p53 and MMR Expression Status

**FIGURE 2:**
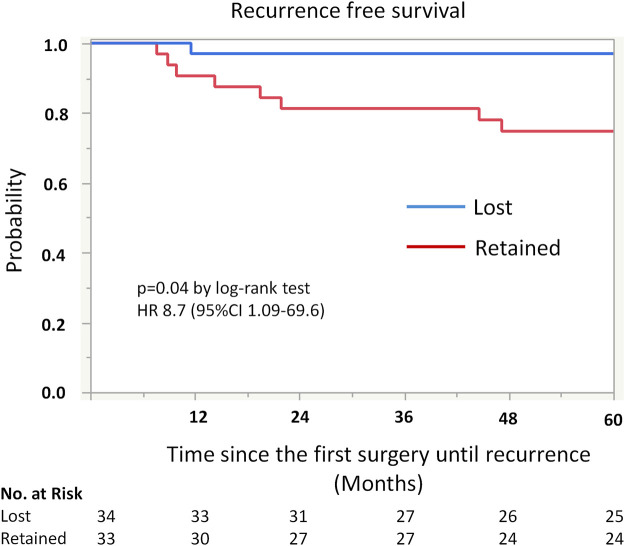
Kaplan-Meier curve for recurrence free survival (RFS) by ARID1A status. The 5-year recurrence-free survival for patients with retained ARID1A expression was 75%. On the other hand, the 5-year recurrence-free survival for those who were ARID1A lose was 97% (*p* = 0.04).

**FIGURE 3:**
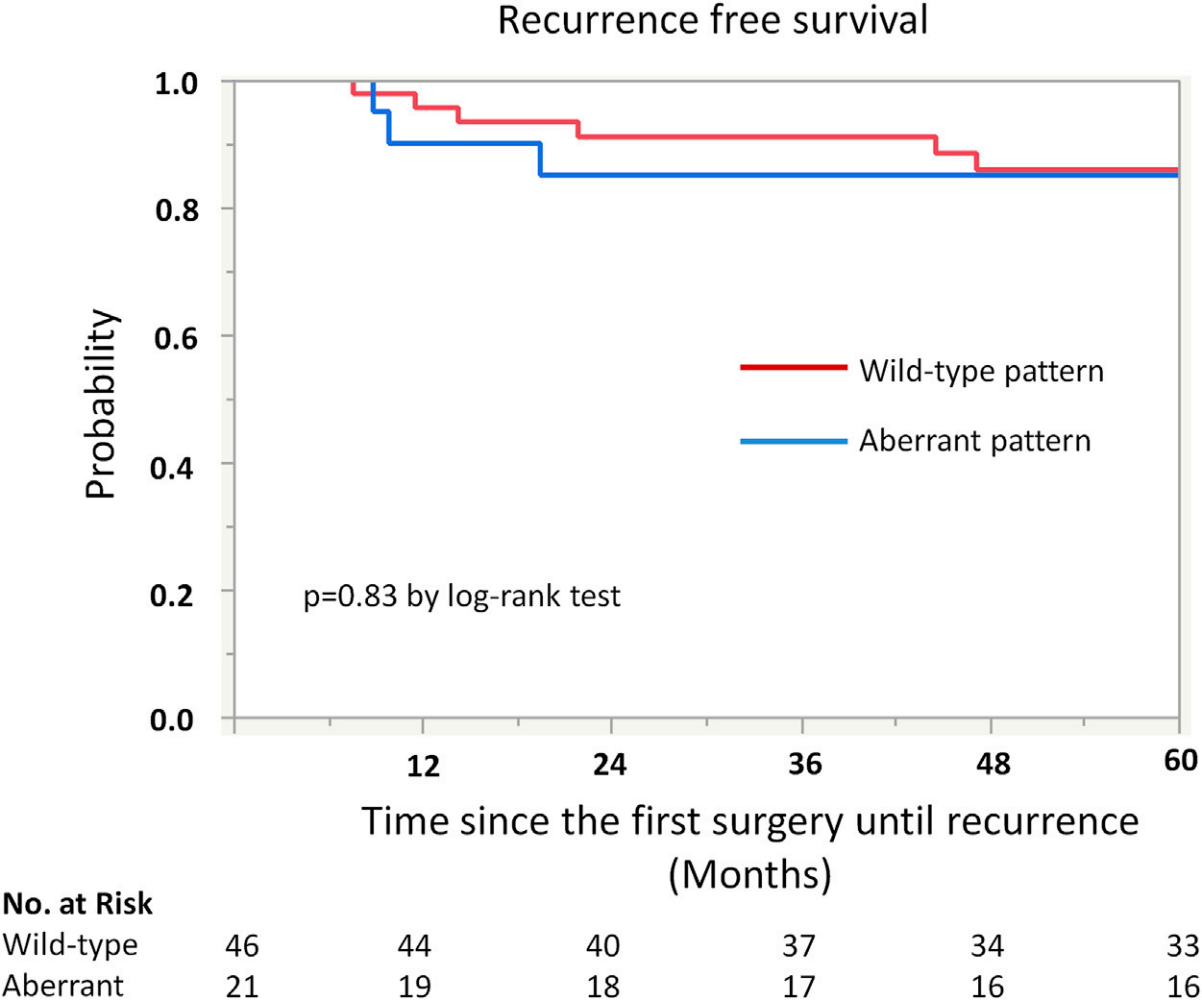
Kaplan-Meier curve for recurrence free survival (RFS) by p53 status.

**FIGURE 4:**
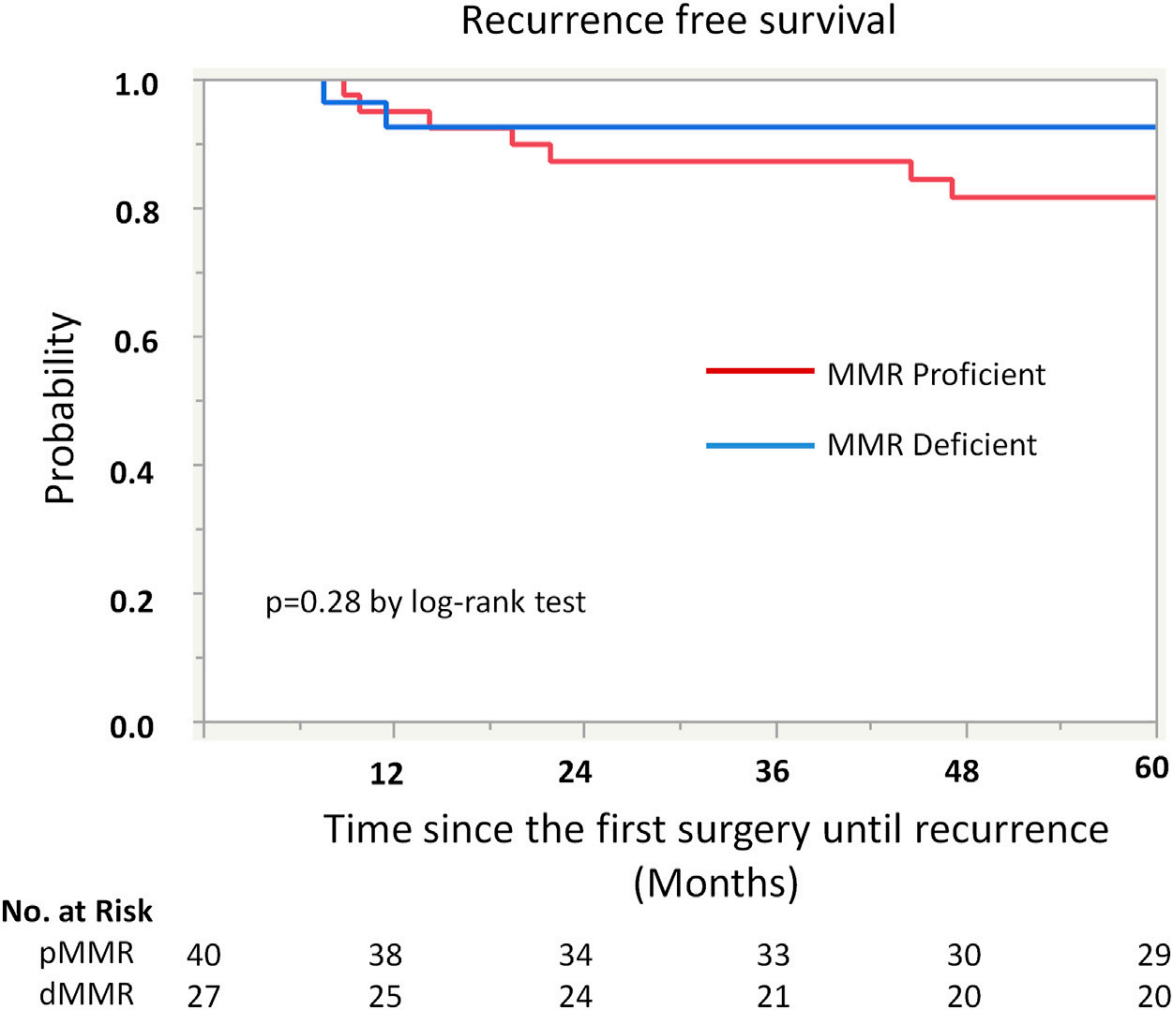
Kaplan-Meier curve for recurrence free survival (RFS) by mismatch repair (MMR) protein expression status.

Kaplan-Meier analysis showed retained ARID1A expression predicting lower RFS in the whole cohort (75% vs. 97%, *p* = 0.04, Table 4; Figure 2). Multivariate Cox regression analysis identified ARID1A status as an independent prognostic factor with a hazard ratio of 8.7 (95% CI 1.09–69.6). No significant correlations were found between recurrence and p53 and MMR expression status (Table 4; Figures 3, 4). Furthermore, survival analysis revealed no significant differences among patients according to ARID1A, p53, and MMR expression status (Supplementary Table S1).

**TABLE 4:** Univariate and multivariate analyses of factors suspected to affect recurrence-free survival.

Variable	Category	N	Univariate analysis	Multivariate analysis		
			(*p* value)	HR	95% CI	*p* value
Age	<65	47	0.07			
	≥65	20				
Stage	I	60	0.82			
	II	7				
Depth of invasion	<1/2	36	0.01	1		
	≥1/2	31		9.9	1.23–79.2	0.03
LVSI	Negative	25	0.1			
	Positive	42				
p53 IHC	Diffuse/Null	21	0.83			
	Wild type	46				
ARID1A IHC	Lost	34	0.01	1		
	Retained	33		8.7	1.09–69.6	0.04
MMR IHC	Proficient	40	0.28			
	Deficient	27				

LVI, lymphovascular space invasion; IHC, immunohistochemistry; ARID1A, AT-rich interaction domain 1A; MMR, mismatch repair.

## Discussion

In the present study, early-stage G3EEC patients had a favorable prognosis. The five-year RFS rates for patients with stage I and II disease were 87.7 and 68.6%, respectively, while the corresponding 5-year OS rates were 93.7 and 85.7%. Loss of ARID1A expression was associated with significantly longer RFS, whereas p53 and MMR proteins expression showed no impact on the five-year RFS and OS rates.

The survival rates for early-stage patients who did not receive any adjuvant therapy were comparable to or better than those reported in previous studies, including patients who received adjuvant therapy (Supplementary Table S2) [2, 4–6, 9, 19]. As the pathological diagnosis of G3EEC is difficult and necessitates interobserver variability [20], a central pathology review is recommended in studies, including cases of high-grade endometrial carcinoma such as G3EEC. In our study, the higher rate of lymph node sampling and detection procedures (90%) yielded more favorable outcomes than previous studies [2, 4].

This study revealed the significant prognostic capabilities of ARID1A expression in early stage G3EEC cases. ARID1A is a recently identified tumor suppressor gene on chromosome 1q36, which participates in forming SWI/SNF chromatin complexes and regulating gene expression. The inactivation of ARID1A promotes the cell cycle and contributes to carcinogenesis [21]. ARID1A mutations were reported in 30–50% of low-grade EECs and 40–60% of high-grade EECs [22, 23]. Furthermore, the loss of ARID1A protein was reported in 29% of low-grade EECs and 39% of high-grade EECs [24]. Mao et al. revealed that ARID1A expression was retained in benign endometrial tissues, but lost in endometrioid intraepithelial neoplasia, low-grade EECs, and high-grade EECs [25]. These observations suggest that ARID1A loss predisposes endometrial carcinogenesis. Moreover, Mao et al. reported a significant increase in the complete loss of ARID1A in the high-grade component of high-grade EECs accompanied with low-grade carcinoma components comprising retained or clonal loss of ARID1A. It indicates that ARID1A loss plays a role in tumor progression [25]. Collectively, ARID1A loss reflected biological aggressiveness of gynecological cancers. However, the prognostic capability of ARID1A loss has not been documented in endometrial cancer.

The prognostic impact of ARID1A loss in cohorts of patients with endometrial cancer of various histological types and/or stages was rarely observed. From a cohort of 535 primary and 77 metastatic endometrial cancer patients, Werner et al. [26] reported significant correlations between ARID1A loss and younger patient age and deeper myometrial invasion, but not survival. In contrast, Zhang et al. [16] observed no associations between ARID1A loss and clinical stage, depth of myometrial invasion, lymph node metastasis, or OS among endometrial carcinoma patients. Notably, Shen et al. [15] demonstrated that ARID1A mutations correlate with favorable survival in endometrial carcinoma using data from The Cancer Genome Atlas. Additionally, they associated ARID1A mutations with better prognosis in patients with microsatellite stable tumors [15]. Interestingly, seven of nine cases with recurrence in our cohort also showed retained ARID1A expression and pMMR phenotype. In addition to MMR deficiency, DNA Polymerase Epsilon and Catalytic Subunit POLE mutation is another favorable prognostic factor, that can be considered in relation to ARID1A loss. Although our study did not include data on mutations, the POLE mutation has been reportedly observed in approximately 10% of endometrial cancers with ARID1A alteration [27]. Therefore, a minor subgroup of cases showing ARID1A loss may have a favorable prognosis attributable to POLE mutations. However, most cases with ARID1A loss do not seem to have a co-existing POLE mutation. Recently, POLE has been reported as one of the putative downstream effectors of ARID1A [28]. In line with this, ARID1A-mutant tumors have been consistently reported as having higher mutation load than that of ARID1A-intact tumors [15, 27]. Collectively, ARID1A loss reportedly promotes mutagenicity of the tumor and may induce a similar phenotype to that of POLE-mutated or MMR-deficient tumors, that may play a key role in endometrial carcinogenesis. Interestingly, co-occurring landscapes of genetic mutations reported in ARID1A-mutated cancers were completely different in early-stage versus advanced-stage cancer [27]. Molecular alterations accompanied with ARID1A loss in each stage of endometrial cancer should be elucidated in future investigations to clarify the role of ARID1A in endometrial carcinogenesis and tumor progression.

Significant prognostic impact of p53 expression status was not observed. An abnormal p53 pattern on IHC staining is an independent poor prognostic factor among endometrial and ovarian cancer patients [29]. Moreover, mutational analysis of G3EEC identified TP53 as an independent prognostic factor for poor RFS [30]. However, this study included patients treated with different types of adjuvant therapy and did not stratify survival analysis by adjuvant treatment. Consequently, its findings cannot be compared with those of the present study. In our study, early-stage G3EEC patients had a favorable prognosis regardless of p53 expression status. According to The Cancer Genome Atlas data on endometrioid endometrial carcinoma, approximately 8% of cases may have POLE mutations, partially including concurrent p53 mutation, and show a favorable prognosis [22]. Since POLE mutation analysis was not performed in the present study, a few cases with concurrent p53 and POLE mutations may compromise the adverse prognostic impact of abnormal p53 expression status. Nevertheless, our finding suggests that IHC analysis of p53 alone was not useful for risk stratification of early-stage G3EEC patients after surgery.

No significant prognostic impact of MMR protein expression was found. Abnormalities in the DNA MMR gene is a hallmark of molecular pathway to carcinogenesis, being observed in 20–40% of cases of endometrial carcinoma [31]. Germline mutations (Lynch syndrome) comprise 3–5% of all defective DNA MMR. The remaining cases are caused by promoter hypermethylation of MLH1, leading to instability of microsatellites [14]. Results associating MMR loss of function with prognosis differ across previous studies [32–36]. While the presence of microsatellite instability was independently associated with a more favorable clinical outcome in endometrial carcinoma [35], other studies demonstrated that young women (younger than 40 or 60 years) with loss of MMR proteins were at risk of high-grade tumors with poor clinical outcomes [32–34]. Moreover, a meta-analysis, including all FIGO tumor stages showed no definitive evidence for a significant association between MMR status and survival outcomes in patients with endometrial cancer [37]. These discrepancies may be attributed to the heterogeneity of the study population. Notably, the current study only included early stage G3EEC patients, who did not receive adjuvant therapy; no significant association was observed between MMR status and prognosis in this population.

Limitations of this study include its single-center retrospective design and the low statistical power attributed to the small number of events. Despite these, this study provided meaningful data regarding the natural clinical course and baseline recurrence risk of early stage G3EEC, by including the largest cohort of pathologically confirmed early-stage G3EEC patients who did not receive adjuvant therapy.

In conclusion, the loss of ARID1A expression favorably prognosticated early-stage G3EEC patients who did not undergo adjuvant therapy. In contrast, p53 and MMR expressions did not show significant prognostic impact on the five-year RFS and OS rates. Although the molecular basis of the relation between ARID1A loss and favorable prognosis remains unclear, the ARID1A expression status may become a useful marker for stratification of adjuvant treatment in early-stage G3EEC patients.

## Data Availability

The datasets presented in this study can be found in online repositories. The names of the repository/repositories and accession number(s) can be found in the article/Supplementary Material.
