# Supplementary material for: Loss of ARID1A Expression as a Favorable Prognostic Factor in Early-Stage Grade 3 Endometrioid Endometrial Carcinoma Patients
Source: Pathol Oncol Res. 2021 Mar 25;27:598550. doi: 10.3389/pore.2021.598550 (PMC8262237; doi:10.3389/pore.2021.598550)
Supplement: Supplementary file 1 [file Table2.DOCX]

**Loss of ARID1A Expression as a Favorable Prognostic Factor in Early-stage Grade 3 Endometrioid Endometrial Carcinoma Patients**

Mayumi Kobayashi Kato^a*^, Hiroshi Yoshida^b*^, Yasuhito Tanase^a^, Masaya Uno^a^, Mitsuya Ishikawa^a^, Tomoyasu Kato^a^

^a^Department of Gynecology, National Cancer Center Hospital, 5-1-1 Tsukiji, Chuo-ku, Tokyo 104-0045, Japan

^b^Department of Diagnostic Pathology, National Cancer Center Hospital, 5-1-1 Tsukiji, Chuo-ku, Tokyo 104-0045, Japan

*Both authors equally contributed to this work.

**Correspondence to**: Mayumi Kobayashi Kato, M.D.

Department of Gynecology, National Cancer Center Hospital

5-1-1 Tsukiji, Chuo-ku, Tokyo 104-0045, Japan

Tel: +81-3-3457-5201, Fax: +81-3-3545-3567, E-mail: [maykobay@ncc.go.jp](mailto:maykobay@ncc.go.jp)

**ORCID:** Mayumi Kobayashi Kato, 0000-0003-4065-0289; Hiroshi Yoshida, 0000-0002-7569-7813; Yasuhito Tanase, 0000-0002-3397-1482; Masaya Uno, 0000-0001-8549-1635; Mitsuya Ishikawa, 0000-0001-8855-2966; Tomoyasu Kato, 0000-0001-9561-1522

**Acknowledgements:** We would like to thank Sachiko Miura, M.T., Toshiko Sakaguchi, M. T., and Chizu Kina, M.T. for technical assistance. We would like to thank Editage ([www.editage.jp](http://www.editage.jp)) for English language editing.

**ABSTRACT**

***Introduction****:* High-risk patients with grade 3 endometrioid endometrial carcinoma (G3EEC) who require adjuvant therapy have not been clearly identified. Therefore, the current study aimed to investigate the prognostic impact of ARID1A, p53, and mismatch repair (MMR) protein expressions, previously reported as prognosticators in some gynecological cancers, in patients with early-stage G3EEC.

***Methods:*** A total of 67 patients with pathologically confirmed early-stage G3EEC diagnosed between 1997 and 2020 were identified; none received adjuvant chemotherapy. The recurrence-free survival (RFS) and overall survival (OS) were estimated using the Kaplan-Meier method and compared with a log-rank test. The protein expressions of ARID1A, p53, and MMR were examined via immunohistochemistry, and the associations between these biomarkers and clinical outcomes were evaluated.

***Results:*** Recurrence was observed in 9 (13%) of the 67 patients with early-stage G3EEC. The respective 5-year RFS and OS rates were 87.7% and 93.7%, and 68.6% and 85.7%, respectively for stages I and II. Multivariate analysis showed significantly longer RFS among patients with ARID1A loss (hazard ratio= 8.7; 95% CI, 1.09–69.6, p=0.04). No significant differences were observed in RFS and OS of patients according to p53 and MMR expression status.

***Conclusion:*** ARID1A expression status was a prognosticator for patients with early-stage G3EEC without adjuvant therapy, whereas p53 and MMR expression status showed no impact on survival outcomes. ARID1A may become a useful biomarker for stratification of adjuvant treatment for early-stage G3EEC patients.

**Keywords:** Endometrioid endometrial carcinoma, grade 3, prognosis, ARID1A, p53, mismatch repair

**INTRODUCTION**

The prevalence of endometrial cancer has been increasing worldwide, with over 380,000 new cases diagnosed in 2018 [1]. In Japan, endometrial cancer is the most commonly observed gynecologic malignancy [2]. Endometrial cancers have been classified as type 1 or type 2 on the basis of the associated risk factors and prognosis [2]. In contrast to type 1, type 2 endometrial cancer is typically not estrogen-driven, and clinically behaves more aggressively.

Grade 3 endometrioid endometrial carcinoma (G3EEC) is conventionally categorized as a type 2 endometrial cancer along with uterine serous carcinoma (USC) and clear cell carcinoma (CCC). Some case series reported similar survival outcomes between G3EEC, USC, and CCC [3-8]. However, in other studies, particularly a retrospective review that used data obtained from the Surveillance, Epidemiology, and End Results Program, patients with G3EEC had a significantly better overall survival (OS) rate than that of patients with USC or CCC [9-11]. Owing to these conflicting results, adjuvant therapy implementation for early-stage G3EEC is controversial; therefore, it is important to clarify the prognostic factors of early-stage G3EEC.

Negative prognostic factors of endometrial carcinoma include non-estrogen dependent tumors (type 2 endometrial cancer), elderly age, and advanced International Federation of Gynecology and Obstetrics (FIGO) stage [12]. Additionally, the expression status of ARID1A, p53, and mismatch repair (MMR) proteins can be independent prognosticators [13,14].

Significant differences were observed between FIGO stages I-IV clear cell and endometrioid subtypes of ovarian and endometrial cancer according to the protein expression status of ARID1A. Several studies reported that *ARID1A* mutations correlated with a favorable survival outcome [15,16]. In addition, aberrant p53 expression was significantly associated with a higher tumor grade and shorter OS [13]. Moreover, MMR deficiency (dMMR) was associated with elderly age, higher tumor grade (G3), and advanced stage (II-IV), whereas *MLH1* promoter hypermethylation predicted a shorter disease-specific survival [14].

However, these studies included all histologic types and stages of endometrial cancer. Only a few studies evaluated the association between prognosis and expression of ARID1A, p53, and MMR, specifically for early-stage G3EEC patients.

In the current study, we retrospectively reviewed patients with early-stage G3EEC and performed immunohistochemistry (IHC) for ARID1A, p53, and MMR proteins. Notably, we could evaluate the baseline recurrence risk in our patients because none of them received adjuvant treatment during the study period due to the lack of compelling evidence in its favor. The study aimed to clarify the prognostic impact of the expression status of ARID1A, p53, and MMR proteins on the outcomes of patients with early-stage G3EEC.

**METHODS**

**Patient Demographic Characteristics**

The study was approved by the Institutional Review Board of the National Cancer Center in Japan (#2016-260) and conducted in accordance with the Declaration of Helsinki. The requirement for informed consent was waived due to the retrospective nature.

Patients with pathologically confirmed stage I and stage II (per the 2008 FIGO classification) G3EEC diagnosed between 1997 and 2020 were identified from the tumor registry database of our institution. All tumors were surgically resected by gynecologic oncologists. All surgical staging procedures, including total hysterectomy, bilateral salpingo-oophorectomy, and pelvic lymph node sampling, were performed via laparotomy. At least two gynecologic pathologists confirmed the histopathological diagnosis of G3EEC and absence of lymph node metastases. Although the Japanese treatment guidelines for endometrial cancer recommend adjuvant chemotherapy for stage IA and IB/II G3EEC, there is no strong evidence supporting this treatment strategy [17]. Thus, the patients included in the present study did not receive adjuvant treatment. The clinicopathological data were collected via a retrospective review of medical charts.

The follow-up for most patients comprised vaginal inspections, Pap smear cytology, and radiological examination including chest radiography every 3–6 months, and computed tomography (CT) of the chest, abdomen, and pelvis every 6 months for the first 2 years. During the 3rd and 4th years of follow-up, patients underwent vaginal examinations, two Pap smears, and annual radiological examinations. After five years of follow-up, patients underwent vaginal inspections and Pap smears annually, and those with suspected G3EEC recurrence underwent CT of the chest to pelvis, magnetic resonance imaging, and histological examination.

**IHC Analysis and Interpretation**

All the surgically resected specimens were fixed in 10% neutral-buffered formalin for 24–72 hours and embedded in paraffin. One representative whole 4-μm-thick section was analyzed using IHC. The following antibodies were used for IHC on the representative slides for each case: anti-p53 (DO7, pre-diluted; Dako, Glostrup, Denmark); anti-ARID1A (polyclonal, 1:2000 dilution; Sigma, St. Louis, MO, USA); anti-hMSH6 (SP93, 1:200 dilution; Spring Bioscience, CA, USA); and anti-hPMS2 (A16-4, 1:200 dilution; Biocare Medical, CA, USA) antibodies. We performed all the IHC tests using a Dako autostainer (Dako, CA, USA) according to the manufacturer’s recommendations. After deparaffinization, tissue sections were stained using the antibodies described above, and then counterstained with hematoxylin.

Aberrant p53 staining pattern was defined as a strong and diffuse nuclear staining pattern (>70% of carcinoma cells) or completely negative (“null pattern”) staining of carcinoma cells with appropriate staining of surrounding non-tumor cells as an internal positive control. A weak and heterogeneous staining pattern of tumor cells was classified as the wild-type pattern.

We assessed ARID1A expression by immunohistochemistry and categorized the results into 3 classes, namely, retained expression (positivity in almost all tumor cells), homogenous loss of expression (negativity in almost all or >90% of tumor cells), and heterogeneous loss of expression (regional negativity with 10-90% of ARID1A-negative tumor cells). Both, homogenous and heterogenous loss of ARID1A expression were regarded as ARID1A loss.

# IHC for PMS2 and MSH6 alone can reportedly replace a four antibody panel (comprising MLH1, MSH2, MSH6, and PMS2) for dMMR screening [18]; therefore, in the present study, MMR-deficient status was defined as the complete loss of nuclear staining for PMS2 and/or MSH6 proteins. Adjacent normal mucosa, stromal cells, and inflammatory cells with intact nuclear staining served as internal positive controls. Representative images of IHC for p53, ARID1A, and MMR proteins are depicted in Figure 1.

**Statistical Analysis**

The follow-up time and time to events were measured from the date of surgery to the last known visit, confirmation of recurrence, or death. Recurrence-free survival (RFS) was defined as the time from the date of surgery to the date of the first recurrence. OS was defined as the time from the date of surgery to the date of death due to any cause. Survival values were estimated using the Kaplan-Meier method and compared with log-rank tests. Independent prognostic factors for RFS were determined using Cox proportional hazards regression models. All analyses were performed using the JMP14 software program (SAS Institute Japan Ltd., Tokyo, Japan).

**RESULTS**

**Patient Demographic Characteristics**

Between 1997 and 2020, a total of 113 patients were diagnosed with G3EEC. All surgical specimens were histologically reviewed, and immunostaining was performed for 110 patients with available specimens.

Among the 113 patients with G3EEC, 67 were identified with FIGO stage I and II disease. None of the patients with early-stage disease received adjuvant treatment; the median age was 58 years (range, 43–80 years). The clinicopathological features of the patients with stage I–II disease are summarized in Table 1.

**Treatment and Prognosis of Patients with Early-stage G3EEC**

All patients with early-stage G3EEC underwent total hysterectomy and bilateral salpingo-oophorectomy. Pelvic lymph node sampling was performed in 60 patients (89.6%). Seven patients did not undergo lymph node sampling due to their advanced age and complications. The median number of removed lymph nodes was 29 (range, 1-58). The median follow-up time for patients with early-stage G3EEC was 65 months (range, 3–182 months). Recurrence was observed in nine patients (13.4%), and the median time to recurrence was 22 months (range, 9–45 months). Recurrences in the vagina, lymph nodes, and other distant sites were observed in one, three, and five patients, respectively.

The clinicopathological features, treatment, and prognosis of the patients with recurrence are summarized in Table 2. The five-year RFS rates for patients with stage I and II disease were 87.7% and 68.6%, respectively. The five-year OS rates for patients with stage I and II disease were 93.7% and 85.7%, respectively. The median RFS and OS were 63.2 months (range, 3–220 months) and 65.6 months (range, 3–227 months), respectively.

**IHC for ARID1A, p53, and MMR Proteins**

The results of IHC staining of early-stage G3EEC are shown in Table 3. An aberrant expression pattern of p53 (overexpression or complete absence) was observed in 21 of 67 cases (31.3%), loss of MMR protein expression was observed in 40.3% of cases, isolated PMS2 loss was observed in 22 of 27 dMMR cases (81.5%), and loss of ARID1A expression was observed in approximately half of the patients (50.7%). There were no significant correlations between ARID1A, p53, and MMR expression status. Moreover, the expression status of these biomarkers was not correlated with clinicopathological features such as tumor size (greatest dimension), depth of myometrial invasion, cervical stromal invasion, lymphovascular invasion, and intraoperative peritoneal cytology.

**Association between Prognosis and the ARID1A, p53 and MMR Expression Status**

Kaplan-Meier analysis showed retained ARID1A expression predicting lower RFS in the whole cohort (75% vs 97%, p=0.04, Table 4, Figure 2). Multivariate Cox regression analysis identified ARID1A status as an independent prognostic factor with a hazard ratio of 8.7 (95% CI 1.09–69.6). No significant correlations were found between recurrence and p53 and MMR expression status (Table 4, Figure 3 and 4). Furthermore, survival analysis revealed no significant differences among patients according to ARID1A, p53, and MMR expression status (Supplementary Table 1).

**DISCUSSION**

In the present study, early-stage G3EEC patients had a favorable prognosis. The five-year RFS rates for patients with stage I and II disease were 87.7% and 68.6%, respectively, while the corresponding 5-year OS rates were 93.7% and 85.7%. Loss of ARID1A expression was associated with significantly longer RFS, whereas p53 and MMR proteins expression showed no impact on the five-year RFS and OS rates.

The survival rates for early-stage patients who did not receive any adjuvant therapy were comparable to or better than those reported in previous studies, including patients who received adjuvant therapy (Supplementary Table 2) [2,4-6,9,19]. As the pathological diagnosis of G3EEC is difficult and necessitates interobserver variability [20], a central pathology review is recommended in studies, including cases of high-grade endometrial carcinoma such as G3EEC. In our study, the higher rate of lymph node sampling and detection procedures (90%) yielded more favorable outcomes than previous studies [2,4].

This study revealed the significant prognostic capabilities of ARID1A expression in early-stage G3EEC cases. *ARID1A* is a recently identified tumor suppressor gene on chromosome 1q36, which participates in forming SWI/SNF chromatin complexes and regulating gene expression. The inactivation of *ARID1A* promotes the cell cycle and contributes to carcinogenesis [21]. *ARID1A* mutations were reported in 30–50% of low-grade EECs and 40–60% of high-grade EECs [22,23]. Furthermore, the loss of ARID1A protein was reported in 29% of low-grade EECs and 39% of high-grade EECs [24]. Mao et al. revealed that ARID1A expression was retained in benign endometrial tissues, but lost in endometrioid intraepithelial neoplasia, low-grade EECs, and high-grade EECs [25]. These observations suggest that ARID1A loss predisposes endometrial carcinogenesis. Moreover, Mao et al. reported a significant increase in the complete loss of ARID1A in the high-grade component of high-grade EECs accompanied with low-grade carcinoma components comprising retained or clonal loss of ARID1A. It indicates that ARID1A loss plays a role in tumor progression [25]. Collectively, ARID1A loss reflected biological aggressiveness of gynecological cancers. However, the prognostic capability of ARID1A loss has not been documented in endometrial cancer.

The prognostic impact of ARID1A loss in cohorts of patients with endometrial cancer of various histological types and/or stages was rarely observed. From a cohort of 535 primary and 77 metastatic endometrial cancer patients, Werner et al. [26] reported significant correlations between ARID1A loss and younger patient age and deeper myometrial invasion, but not survival. In contrast, Zhang et al. [16] observed no associations between ARID1A loss and clinical stage, depth of myometrial invasion, lymph node metastasis, or OS among endometrial carcinoma patients. Notably, Shen et al. [15] demonstrated that *ARID1A* mutations correlate with favorable survival in endometrial carcinoma using data from The Cancer Genome Atlas. Additionally, they associated *ARID1A* mutations with better prognosis in patients with microsatellite stable tumors [15]. Interestingly, seven of nine cases with recurrence in our cohort also showed retained ARID1A expression and pMMR phenotype. In addition to MMR deficiency, DNA Polymerase Epsilon and Catalytic Subunit *POLE* mutation is another favorable prognostic factor, that can be considered in relation to ARID1A loss. Although our study did not include data on mutations, the *POLE* mutation has been reportedly observed in approximately 10% of endometrial cancers with *ARID1A* alteration [27]. Therefore, a minor subgroup of cases showing ARID1A loss may have a favorable prognosis attributable to *POLE* mutations. However, most cases with ARID1A loss do not seem to have a co-existing *POLE* mutation. Recently, *POLE* has been reported as one of the putative downstream effectors of *ARID1A* [28]. In line with this, ARID1A-mutant tumors have been consistently reported as having higher mutation load than that of ARID1A-intact tumors [27,15]. Collectively, ARID1A loss reportedly promotes mutagenicity of the tumor and may induce a similar phenotype to that of *POLE*-mutated or MMR-deficient tumors, that may play a key role in endometrial carcinogenesis. Interestingly, co-occurring landscapes of genetic mutations reported in *ARID1A*-mutated cancers were completely different in early-stage versus advanced-stage cancer [27]. Molecular alterations accompanied with ARID1A loss in each stage of endometrial cancer should be elucidated in future investigations to clarify the role of ARID1A in endometrial carcinogenesis and tumor progression.

Significant prognostic impact of p53 expression status was not observed. An abnormal p53 pattern on IHC staining is an independent poor prognostic factor among endometrial and ovarian cancer patients [29]. Moreover, mutational analysis of G3EEC identified TP53 as an independent prognostic factor for poor RFS [30]. However, this study included patients treated with different types of adjuvant therapy and did not stratify survival analysis by adjuvant treatment. Consequently, its findings cannot be compared with those of the present study. In our study, early-stage G3EEC patients had a favorable prognosis regardless of p53 expression status. According to The Cancer Genome Atlas data on endometrioid endometrial carcinoma, approximately 8% of cases may have *POLE* mutations, partially including concurrent *p53* mutation, and show a favorable prognosis [22]. Since *POLE* mutation analysis was not performed in the present study, a few cases with concurrent *p53* and *POLE* mutations may compromise the adverse prognostic impact of abnormal p53 expression status. Nevertheless, our finding suggests that IHC analysis of p53 alone was not useful for risk stratification of early-stage G3EEC patients after surgery.

No significant prognostic impact of MMR protein expression was found. Abnormalities in the DNA MMR gene is a hallmark of molecular pathway to carcinogenesis, being observed in 20–40% of cases of endometrial carcinoma [31]. Germline mutations (Lynch syndrome) comprise 3–5% of all defective DNA MMR. The remaining cases are caused by promoter hypermethylation of *MLH1*, leading to instability of microsatellites [14]. Results associating MMR loss of function with prognosis differ across previous studies [32-36]. While the presence of microsatellite instability was independently associated with a more favorable clinical outcome in endometrial carcinoma [35], other studies demonstrated that young women (younger than 40 years or 60 years) with loss of MMR proteins were at risk of high-grade tumors with poor clinical outcomes [32-34]. Moreover, a meta-analysis, including all FIGO tumor stages showed no definitive evidence for a significant association between MMR status and survival outcomes in patients with endometrial cancer [37]. These discrepancies may be attributed to the heterogeneity of the study population. Notably, the current study only included early-stage G3EEC patients, who did not receive adjuvant therapy; no significant association was observed between MMR status and prognosis in this population.

Limitations of this study include its single-center retrospective design and the low statistical power attributed to the small number of events. Despite these, this study provided meaningful data regarding the natural clinical course and baseline recurrence risk of early-stage G3EEC, by including the largest cohort of pathologically confirmed early-stage G3EEC patients who did not receive adjuvant therapy.

In conclusion, the loss of ARID1A expression favorably prognosticated early-stage G3EEC patients who did not undergo adjuvant therapy. In contrast, p53 and MMR expressions did not show significant prognostic impact on the five-year RFS and OS rates. Although the molecular basis of the relation between ARID1A loss and favorable prognosis remains unclear, the ARID1A expression status may become a useful marker for stratification of adjuvant treatment in early-stage G3EEC patients.

**Declarations**

**Funding:** This work was supported by JSPS KAKENHI Grant Numbers JP20K18207 and JP19K16572.

**Conflict of interest**: The authors declare that they have no conflict of interest.

**Ethics approval:** The study was approved by the Institutional Review Board of the National Cancer Center in Japan (#2016-260) and conducted in accordance with the Declaration of Helsinki.

**Consent to participate:** Informed consent was waived.

**Consent for publication:** Not applicable.

**Availability of data and material:** All data generated or analyzed during this study are included in this published article and its supplementary information files.

**Code availability:** Not applicable.

**Authors’ contributions:** All authors contributed to the study conception and design. Material preparation, data collection and analysis were performed by Mayumi Kobayashi Kato and Hiroshi Yoshida. The first draft of the manuscript was written by Mayumi Kobayashi Kato and all authors commented on previous versions of the manuscript. All authors read and approved the final manuscript.

**REFERENCES**

1. Siegel RL, Miller KD, Jemal A (2019) Cancer statistics, 2019. CA Cancer J Clin 69 (1):7-34. doi:10.3322/caac.21551

2. McGunigal M, Liu J, Kalir T, Chadha M, Gupta V (2017) Survival Differences Among Uterine Papillary Serous, Clear Cell and Grade 3 Endometrioid Adenocarcinoma Endometrial Cancers: A National Cancer Database Analysis. Int J Gynecol Cancer 27 (1):85-92. doi:10.1097/IGC.0000000000000844

3. Voss MA, Ganesan R, Ludeman L, McCarthy K, Gornall R, Schaller G, Wei W, Sundar S (2012) Should grade 3 endometrioid endometrial carcinoma be considered a type 2 cancer-a clinical and pathological evaluation. Gynecol Oncol 124 (1):15-20. doi:10.1016/j.ygyno.2011.07.030

4. Creasman WT, Kohler MF, Odicino F, Maisonneuve P, Boyle P (2004) Prognosis of papillary serous, clear cell, and grade 3 stage I carcinoma of the endometrium. Gynecol Oncol 95 (3):593-596. doi:10.1016/j.ygyno.2004.08.019

5. Alektiar KM, McKee A, Lin O, Venkatraman E, Zelefsky MJ, McKee B, Hoskins WJ, Barakat RR (2002) Is there a difference in outcome between stage I-II endometrial cancer of papillary serous/clear cell and endometrioid FIGO Grade 3 cancer? Int J Radiat Oncol Biol Phys 54 (1):79-85. doi:10.1016/s0360-3016(02)02913-9

6. Cirisano FD, Jr., Robboy SJ, Dodge RK, Bentley RC, Krigman HR, Synan IS, Soper JT, Clarke-Pearson DL (2000) The outcome of stage I-II clinically and surgically staged papillary serous and clear cell endometrial cancers when compared with endometrioid carcinoma. Gynecol Oncol 77 (1):55-65. doi:10.1006/gyno.2000.5737

7. Soslow RA, Bissonnette JP, Wilton A, Ferguson SE, Alektiar KM, Duska LR, Oliva E (2007) Clinicopathologic analysis of 187 high-grade endometrial carcinomas of different histologic subtypes: similar outcomes belie distinctive biologic differences. Am J Surg Pathol 31 (7):979-987. doi:10.1097/PAS.0b013e31802ee494

8. Alvarez T, Miller E, Duska L, Oliva E (2012) Molecular profile of grade 3 endometrioid endometrial carcinoma: is it a type I or type II endometrial carcinoma? Am J Surg Pathol 36 (5):753-761. doi:10.1097/PAS.0b013e318247b7bb

9. Hamilton CA, Cheung MK, Osann K, Chen L, Teng NN, Longacre TA, Powell MA, Hendrickson MR, Kapp DS, Chan JK (2006) Uterine papillary serous and clear cell carcinomas predict for poorer survival compared to grade 3 endometrioid corpus cancers. Br J Cancer 94 (5):642-646. doi:10.1038/sj.bjc.6603012

10. Alkushi A, Kobel M, Kalloger SE, Gilks CB (2010) High-grade endometrial carcinoma: serous and grade 3 endometrioid carcinomas have different immunophenotypes and outcomes. Int J Gynecol Pathol 29 (4):343-350. doi:10.1097/PGP.0b013e3181cd6552

11. Boruta DM, 2nd, Gehrig PA, Groben PA, Bae-Jump V, Boggess JF, Fowler WC, Jr., Van Le L (2004) Uterine serous and grade 3 endometrioid carcinomas: is there a survival difference? Cancer 101 (10):2214-2221. doi:10.1002/cncr.20645

12. Saso S, Chatterjee J, Georgiou E, Ditri AM, Smith JR, Ghaem-Maghami S (2011) Endometrial cancer. BMJ 343:d3954. doi:10.1136/bmj.d3954

13. Heckl M, Schmoeckel E, Hertlein L, Rottmann M, Jeschke U, Mayr D (2018) The ARID1A, p53 and ss-Catenin statuses are strong prognosticators in clear cell and endometrioid carcinoma of the ovary and the endometrium. PLoS One 13 (2):e0192881. doi:10.1371/journal.pone.0192881

14. Pasanen A, Loukovaara M, Butzow R (2020) Clinicopathological significance of deficient DNA mismatch repair and MLH1 promoter methylation in endometrioid endometrial carcinoma. Mod Pathol. doi:10.1038/s41379-020-0501-8

15. Shen J, Ju Z, Zhao W, Wang L, Peng Y, Ge Z, Nagel ZD, Zou J, Wang C, Kapoor P, Ma X, Ma D, Liang J, Song S, Liu J, Samson LD, Ajani JA, Li GM, Liang H, Shen X, Mills GB, Peng G (2018) ARID1A deficiency promotes mutability and potentiates therapeutic antitumor immunity unleashed by immune checkpoint blockade. Nat Med 24 (5):556-562. doi:10.1038/s41591-018-0012-z

16. Zhang ZM, Xiao S, Sun GY, Liu YP, Zhang FH, Yang HF, Li J, Qiu HB, Liu Y, Zhang C, Kang S, Shan BE (2014) The clinicopathologic significance of the loss of BAF250a (ARID1A) expression in endometrial carcinoma. Int J Gynecol Cancer 24 (3):534-540. doi:10.1097/IGC.0000000000000092

17. Susumu N, Sagae S, Udagawa Y, Niwa K, Kuramoto H, Satoh S, Kudo R, Japanese Gynecologic Oncology G (2008) Randomized phase III trial of pelvic radiotherapy versus cisplatin-based combined chemotherapy in patients with intermediate- and high-risk endometrial cancer: a Japanese Gynecologic Oncology Group study. Gynecol Oncol 108 (1):226-233. doi:10.1016/j.ygyno.2007.09.029

18. Hall G, Clarkson A, Shi A, Langford E, Leung H, Eckstein RP, Gill AJ (2010) Immunohistochemistry for PMS2 and MSH6 alone can replace a four antibody panel for mismatch repair deficiency screening in colorectal adenocarcinoma. Pathology 42 (5):409-413. doi:10.3109/00313025.2010.493871

19. Rasool N, Fader AN, Seamon L, Neubauer NL, Shahin FA, Alexander HA, Moore K, Moxley K, Secord AA, Kunos C, Rose PG, O'Malley DM (2010) Stage I, grade 3 endometrioid adenocarcinoma of the endometrium: an analysis of clinical outcomes and patterns of recurrence. Gynecol Oncol 116 (1):10-14. doi:10.1016/j.ygyno.2009.10.043

20. Thomas S, Hussein Y, Bandyopadhyay S, Cote M, Hassan O, Abdulfatah E, Alosh B, Guan H, Soslow RA, Ali-Fehmi R (2016) Interobserver Variability in the Diagnosis of Uterine High-Grade Endometrioid Carcinoma. Arch Pathol Lab Med 140 (8):836-843. doi:10.5858/arpa.2015-0220-OA

21. Wang X, Nagl NG, Wilsker D, Van Scoy M, Pacchione S, Yaciuk P, Dallas PB, Moran E (2004) Two related ARID family proteins are alternative subunits of human SWI/SNF complexes. Biochem J 383 (Pt 2):319-325. doi:10.1042/BJ20040524

22. Cancer Genome Atlas Research N, Kandoth C, Schultz N, Cherniack AD, Akbani R, Liu Y, Shen H, Robertson AG, Pashtan I, Shen R, Benz CC, Yau C, Laird PW, Ding L, Zhang W, Mills GB, Kucherlapati R, Mardis ER, Levine DA (2013) Integrated genomic characterization of endometrial carcinoma. Nature 497 (7447):67-73. doi:10.1038/nature12113

23. McConechy MK, Ding J, Cheang MC, Wiegand K, Senz J, Tone A, Yang W, Prentice L, Tse K, Zeng T, McDonald H, Schmidt AP, Mutch DG, McAlpine JN, Hirst M, Shah SP, Lee CH, Goodfellow PJ, Gilks CB, Huntsman DG (2012) Use of mutation profiles to refine the classification of endometrial carcinomas. J Pathol 228 (1):20-30. doi:10.1002/path.4056

24. Wiegand KC, Lee AF, Al-Agha OM, Chow C, Kalloger SE, Scott DW, Steidl C, Wiseman SM, Gascoyne RD, Gilks B, Huntsman DG (2011) Loss of BAF250a (ARID1A) is frequent in high-grade endometrial carcinomas. J Pathol 224 (3):328-333. doi:10.1002/path.2911

25. Mao TL, Ardighieri L, Ayhan A, Kuo KT, Wu CH, Wang TL, Shih Ie M (2013) Loss of ARID1A expression correlates with stages of tumor progression in uterine endometrioid carcinoma. The American journal of surgical pathology 37 (9):1342-1348. doi:10.1097/PAS.0b013e3182889dc3

26. Werner HM, Berg A, Wik E, Birkeland E, Krakstad C, Kusonmano K, Petersen K, Kalland KH, Oyan AM, Akslen LA, Trovik J, Salvesen HB (2013) ARID1A loss is prevalent in endometrial hyperplasia with atypia and low-grade endometrioid carcinomas. Mod Pathol 26 (3):428-434. doi:10.1038/modpathol.2012.174

27. Jiang T, Chen X, Su C, Ren S, Zhou C (2020) Pan-cancer analysis of ARID1A Alterations as Biomarkers for Immunotherapy Outcomes. J Cancer 11 (4):776-780. doi:10.7150/jca.41296

28. Kurz L, Miklyaeva A, Skowron MA, Overbeck N, Poschmann G, Becker T, Eul K, Kurz T, Schonberger S, Calaminus G, Stuhler K, Dykhuizen E, Albers P, Nettersheim D (2020) ARID1A Regulates Transcription and the Epigenetic Landscape via POLE and DMAP1 while ARID1A Deficiency or Pharmacological Inhibition Sensitizes Germ Cell Tumor Cells to ATR Inhibition. Cancers (Basel) 12 (4). doi:10.3390/cancers12040905

29. Lee YH, Heo JH, Kim TH, Kang H, Kim G, Kim J, Cho SH, An HJ (2011) Significance of cell cycle regulatory proteins as malignant and prognostic biomarkers in ovarian epithelial tumors. Int J Gynecol Pathol 30 (3):205-217. doi:10.1097/PGP.0b013e3182063e71

30. Bosse T, Nout RA, McAlpine JN, McConechy MK, Britton H, Hussein YR, Gonzalez C, Ganesan R, Steele JC, Harrison BT, Oliva E, Vidal A, Matias-Guiu X, Abu-Rustum NR, Levine DA, Gilks CB, Soslow RA (2018) Molecular Classification of Grade 3 Endometrioid Endometrial Cancers Identifies Distinct Prognostic Subgroups. The American journal of surgical pathology 42 (5):561-568. doi:10.1097/pas.0000000000001020

31. Karamurzin Y, Rutgers JK (2009) DNA mismatch repair deficiency in endometrial carcinoma. Int J Gynecol Pathol 28 (3):239-255. doi:10.1097/PGP.0b013e31818d8fe6

32. Garg K, Shih K, Barakat R, Zhou Q, Iasonos A, Soslow RA (2009) Endometrial carcinomas in women aged 40 years and younger: tumors associated with loss of DNA mismatch repair proteins comprise a distinct clinicopathologic subset. Am J Surg Pathol 33 (12):1869-1877. doi:10.1097/PAS.0b013e3181bc9866

33. Shih KK, Garg K, Levine DA, Kauff ND, Abu-Rustum NR, Soslow RA, Barakat RR (2011) Clinicopathologic significance of DNA mismatch repair protein defects and endometrial cancer in women 40years of age and younger. Gynecol Oncol 123 (1):88-94. doi:10.1016/j.ygyno.2011.06.005

34. Grzankowski KS, Shimizu DM, Kimata C, Black M, Terada KY (2012) Clinical and pathologic features of young endometrial cancer patients with loss of mismatch repair expression. Gynecol Oncol 126 (3):408-412. doi:10.1016/j.ygyno.2012.05.019

35. Black D, Soslow RA, Levine DA, Tornos C, Chen SC, Hummer AJ, Bogomolniy F, Olvera N, Barakat RR, Boyd J (2006) Clinicopathologic significance of defective DNA mismatch repair in endometrial carcinoma. J Clin Oncol 24 (11):1745-1753. doi:10.1200/JCO.2005.04.1574

36. Zighelboim I, Goodfellow PJ, Gao F, Gibb RK, Powell MA, Rader JS, Mutch DG (2007) Microsatellite instability and epigenetic inactivation of MLH1 and outcome of patients with endometrial carcinomas of the endometrioid type. J Clin Oncol 25 (15):2042-2048. doi:10.1200/JCO.2006.08.2107

37. Diaz-Padilla I, Romero N, Amir E, Matias-Guiu X, Vilar E, Muggia F, Garcia-Donas J (2013) Mismatch repair status and clinical outcome in endometrial cancer: a systematic review and meta-analysis. Crit Rev Oncol Hematol 88 (1):154-167. doi:10.1016/j.critrevonc.2013.03.002

Table 1. Characteristics of patients with early-stage grade 3 endometrioid endometrial carcinoma

| Characteristic | | No (%) (n=67) |
| --- | --- | --- |
| Stage (FIGO 2008) | IA | 35 (52) |
|  | IB | 25 (37) |
|  | II | 7 (10) |
| Age, years | <65 | 47 (70) |
|  | ≥65 | 20 (30) |
| BMI | <30 | 62 (93) |
|  | ≥30 | 5 (7) |
| Depth of invasion | Endometrium | 5 (7) |
|  | Myometrium <50% | 31 (46) |
|  | Myometrium ≥50% | 31 (46) |
| Cervical stromal involvement | Present | 7 (10) |
|  | Absent | 60 (90) |
| LVSI | Present | 42 (63) |
|  | Absent | 25 (37) |
| Pelvic cytology | Positive | 8 (12) |
|  | Negative | 59 (88) |

BMI, body mass index; FIGO, International Federation of Gynecology and Obstetrics; LVSI, lymphovascular space invasion

Table 2. Clinicopathological characteristics, treatment, and outcomes of patients with recurrence of early-stage grade 3 endometrioid endometrial carcinoma

| Case | Age | Stage | Tumor  size  (mm) | Depth of invasion | LVSI | p53 IHC | ARID1A  IHC | MMR status | site of recurrence | Treatment for recurrence | DFS (mo) | Status at last  follow-up |
| --- | --- | --- | --- | --- | --- | --- | --- | --- | --- | --- | --- | --- |
| 1 | 63 | IA | 33 | <1/2 | Present | Wild type | lost (homo) | deficient (PMS2) | bone | RT+Chemotherapy | 12 | LOF,17months |
| 2 | 57 | II | 35 | ≥1/2 | Absent | Wild type | retained | proficient | lung(44mo), abdominal mass(96mo) | Surgery+Chemotherapy | 44 | NED, 182months |
| 3 | 65 | IB | 120 | ≥1/2 | Present | Wild type | retained | proficient | PAN | Surgery+Chemotherapy | 13 | NED, 69months |
| 4 | 70 | II | 66 | ≥1/2 | Present | Diffuse | retained | proficient | PLN | MPA | 17 | DOD, 32months |
| 5 | 67 | IB | 40 | ≥1/2 | Present | Wild type | retained | proficient | peritoneum+pleura | Chemotherapy | 21 | DOD, 40months |
| 6 | 69 | IB | 15 | ≥1/2 | Present | Wild type | retained | proficient | lung | Chemotherapy | 27 | DOD, 47months |
| 7 | 54 | IB | 70 | ≥1/2 | Present | Wild type | retained | deficient (PMS2) | Vagina | RT | 6 | NED, 45months |
| 8 | 54 | IB | 70 | ≥1/2 | Present | Diffuse | retained | proficient | Liver, PAN | Chemotherapy | 10 | NED, 23months |
| 9 | 71 | IB | 40 | ≥1/2 | Present | Diffuse | retained | proficient | lung | Chemotherapy | 9 | AWD, 9months |

LVSI, lymphovascular space invasion; ARID1A, AT-rich interaction domain 1A; MMR, mismatch repair; DFS, disease-free survival; LOF, lost to follow-up; NED, no evidence of disease; PAN, para-aortic lymph node; PLN, pelvic lymph node; MPA, medroxyprogesterone acetate; DOD, died of disease

Table 3. Number of patients showing immunohistochemical staining of early-stage grade 3 endometrioid endometrial carcinoma

| Marker | Staining pattern | No (%) (n=67) |
| --- | --- | --- |
| p53 | Aberrant pattern | 21 (31) |
|  | Diffuse pattern | 20 (29) |
|  | Null pattern | 1 (1) |
|  | Wild-type pattern | 46 (69) |
|  |  |  |
| ARID1A | Retained | 33 (49) |
|  | Lost | 34 (51) |
|  | Homogenous pattern | 22 |
|  | Heterogenous pattern | 12 |
|  |  |  |
| MMR | Proficient | 40 (60) |
|  | Deficient | 27 (40) |
|  | PMS2 | 22 |
|  | MSH6 | 5 |

ARID1A: AT-rich interaction domain 1A, MMR: mismatch repair

Table 4. Univariate and multivariate analyses of factors suspected to affect recurrence-free survival

| Variable | Category | N | Univariate analysis | Multivariate analysis | | |
| --- | --- | --- | --- | --- | --- | --- |
|  |  |  | (p value) | HR | 95%CI | pValue |
| Age | <65 | 47 | 0.07 |  |  |  |
|  | ≥65 | 20 |  |  |  |  |
| Stage | I | 60 | 0.82 |  |  |  |
|  | II | 7 |  |  |  |  |
| Depth of invasion | <1/2 | 36 | 0.01 | 1 |  |  |
|  | ≥1/2 | 31 |  | 9.9 | 1.23-79.2 | 0.03 |
| LVSI | Negative | 25 | 0.1 |  |  |  |
|  | Positive | 42 |  |  |  |  |
| p53 IHC | Diffuse/Null | 21 | 0.83 |  |  |  |
|  | Wild type | 46 |  |  |  |  |
| ARID1A IHC | Lost | 34 | 0.01 | 1 |  |  |
|  | Retained | 33 |  | 8.7 | 1.09-69.6 | 0.04 |
| MMR IHC | Proficient | 40 | 0.28 |  |  |  |
|  | Deficient | 27 |  |  |  |  |

LVI, lymphovascular space invasion; IHC, immunohistochemistry; ARID1A, AT-rich interaction domain 1A; MMR, mismatch repair

**FIGURE LEGENDS**

**Figure 1.** Immunohistochemistry staining results of grade 3 endometrioid endometrial carcinoma. The tumor is dominantly composed of solid nests (a, b). TP53 showing wild-type (c), diffuse strong (d), and null patterns (e). ARID1A showing intact staining (f), complete loss (g), and partial loss (h). PMS2 nuclear staining is retained (i) and lost (j). MSH6 staining is lost (k).

**Figure 2.** Kaplan-Meier curve for recurrence free survival (RFS) by ARID1A status. The 5-year recurrence-free survival for patients with retained ARID1A expression was 75%. On the other hand, the 5-year recurrence-free survival for those who were ARID1A lose was 97% (p=0.04)

**Figure 3.** Kaplan-Meier curve for recurrence free survival (RFS) by p53 status.

**Figure 4.** Kaplan-Meier curve for recurrence free survival (RFS) by mismatch repair (MMR) protein expression status.
